# Emerging Infectious Diseases in Mongolia

**DOI:** 10.3201/eid0912.020520

**Published:** 2003-12

**Authors:** John R. Ebright, Togoo Altantsetseg, Ravdan Oyungerel

**Affiliations:** * Wayne State University School of Medicine, Detroit, Michigan, USA; † National Medical University of Mongolia, Ulaanbaatar, Mongolia; ‡ National Research Center for Infectious Diseases, Ulaanbaatar, Mongolia

**Keywords:** Mongolia, infectious diseases, emerging infections

## Abstract

Since 1990, Mongolia’s health system has been in transition. Impressive gains have been accomplished through a national immunization program, which was instituted in 1991. Nevertheless, the country continues to confront four major chronic infections: hepatitis B and C, brucellosis, tuberculosis, and sexually transmitted diseases (STDs). As of 2001, only two cases of HIV infections had been detected in Mongolia, but concern grows that the rate will increase along with the rising rates of STDs and increase in tourism. Other infectious diseases of importance in Mongolia include echinococcus, plague, tularemia, anthrax, foot-and-mouth, and rabies.

Mongolia, situated in Central Asia between Russia and China, has a population of 2.5 million people and an average population density of 1.5 persons per sq. km. Twenty-seven percent of the country’s total population lives in the capital city, Ulaanbaatar. The country is divided into 21 administrative provinces called *aimags* with medical care delivered in each province through a three-tiered system; referral to any of the university joint hospitals in Ulaanbaatar is possible. As of 1997, Mongolia had 25 physicians and 78 hospital beds per 10,000 population, one of the highest ratios in Asia (*1*–*3*).

Major political and healthcare changes began in 1990, when Mongolia ceased to be under Soviet control and stopped receiving developmental aid as one of the Eastern block satellites (*2*). Since that time, its economy has been changing from a centrally planned socialist system to a free market economy with healthcare delivery reflecting that transition (*1*,*2*,*4*–*6*).

Although progress is being made, Mongolia continues to struggle with poor transportation and communication and limited material (including laboratory) facilities. Financial difficulties remain a major challenge as the country seeks to develop economic self-sufficiency and deliver modern health care to its people (*1*).

This report results from two onsite visits by one of the authors (JRE) in September 1999 and May 2001 which allowed for extensive discussions with infectious diseases physicians of the National Research Center for Infectious Diseases (NRCID) and the National Medical University of Mongolia. In addition, he was able to attend hospital ward rounds and conduct patient examinations at NRCID, the Central Hospital of Oncology, First Clinical Hospital, and Third Clinical Hospital in Ulaanbaatar and to review the laboratory facilities in those hospitals.

NRCID serves as the major university hospital and referral center for tuberculosis (TB) and chronic infectious diseases in Mongolia. It is situated on 7 acres of land in south Ulaanbaatar and consists of 23 brick buildings constructed in 1985 by the Soviet Union. Each building is free-standing and requires health providers to walk outside in order to go from building to building (an important point considering Ulaanbaatar’s severe and prolonged winters). As a government facility, NRCID is managed and maintained through government funds. Patients are provided care regardless of their ability to pay, and full-time staff physicians receive government salaries.

The data in the following sections of this report were derived from three sources. The first represents the annual cumulative number of specific infectious diseases, which have a high public health impact in Mongolia. These data were collected monthly throughout the country by healthcare personnel in all rural districts and provinces, as well as in Ulaanbaatar, and reported to the Ministry of Health and Social Welfare. The annual cumulative data for each disease were summarized and published for the medical community and general public by the National Statistical Office (Table). The second source is derived from the personal discussions held with leaders in public health and microbiology as well as infectious disease clinicians and faculty at NRCID, the National Medical University, and other hospitals in Ulaanbaatar. The third consists of recently published reports dealing with infectious diseases and public health issues in Mongolia during the past 10 years and identified through Medline search.

**Table:** Annual cumulative number of reportable infectious diseases by year, Mongolia^a^

Disease	Y					
	1990	1995	1996	1997	1998	1999
Tuberculosis	1,664	2,543	3,104	2,723	2,806	3,221
Viral hepatitis	14,278	7,877	8,198	9,394	8,042	5,249
Brucellosis	—	850	1,158	1,122	1,308	1,482
Syphilis	705	718	810	1,291	1,329	1,093
Gonorrhea	2,234	3,308	3,274	2,934	3,486	2,207
Salmonella	866	360	323	256	239	243
Shigella	1,930	1,589	2,294	2,146	1,261	1,383
Measles	296	558	123	4	8	10
Mumps	240	255	436	736	1,287	426
Meningococcal meningitis	776	2,781	881	533	303	242
Chickenpox	810	401	386	253	375	297

^a^(ref. *9*, modified)

## TB

A separate building at NRCID is dedicated to management of patients with TB. Divided by separate floors into pediatric, adult, and surgical units, the TB hospital houses approximately 230 patients and serves as the primary referral and treatment center for Mongolia. Diagnosis is made by clinical symptoms, chest x-ray, Mantoux skin test, and positive acid-fast smears of clinical specimens. Availability of culture and susceptibility data appears to be inconsistent but is expected to improve when the National Tuberculosis Central Laboratory is moved on site at NRCID.

Staff physicians treat all patients with isoniazid, rifampin, pyrazinamide, and ethambutol (or streptomycin) for the first 2 of a standard 6-month treatment course and administer treatment by direct observation (DOT). This treatment is an improvement compared with the TB program of the mid-1990s, when only 29% of patients received a four-drug treatment course (7). Current data from the pediatric unit indicate that 93% of patients became smear-negative after the initial 2-month DOT program compared with 100% 5 years ago. This finding is similar to results (92% smear-negative) reported from a pilot study that used the same antimicrobial drugs and DOT program in eastern Mongolia in 1999 (*8*). On becoming smear-negative, the patients are discharged to their homes and complete the remainder of their 6-month program with isoniazid and rifampin alone. Both the central hospital and regional clinics cooperate to oversee the completion of therapy after discharge and to ensure that contact tracing is performed. Anti-TB medication is available only through the central TB hospital and regional clinics and not from private pharmacies. In general, no second-line anti-TB drugs, except ciprofloxacin, are available in Mongolia.

National statistics show the lowest incidence of TB in the past 10 years occurred during 1990 to 1994 (1,664 cases, 1990) with a marked increase by 1995 continuing through 1999 (3,104 cases, 1996; 3,221 cases, 1999) (*9*). The increase probably represents a reporting bias resulting from a relative lack of evaluation and treatment available during the first few years after Mongolia was no longer under Soviet control and until the national TB program was established in 1994 (*7*). Data from the National Tuberculosis Central Laboratory in Ulaanbaatar characterize the 3,221 cases that occurred in 1999 as follows: pulmonary, 2,280 cases (70%) with 1,513 diagnosed by positive sputum smears and 767 (33% of total pulmonary cases) diagnosed clinically in spite of negative smears; and extrapulmonary, 941 cases. Susceptibility data available from the same source indicate primary resistance to isoniazid is >10%; streptomycin resistance is >10%; any resistance is 29%; and multiple-resistant isolates is 1.1% (N. Naranbat, pers. commun.).

## Viral Hepatitis

Hospital directors of NRCID as well as senior infectious diseases faculty of the National Medical University agree that the most common reportable infection in Mongolia is viral hepatitis. This fact is still true even though the incidence has decreased from 14,278 cases in 1990 to 5,249 cases in 1999 (*9*). Information regarding frequencies of specific types of viral hepatitis is more difficult to obtain. Serologic testing for hepatitis C has been readily available only since 1999. One physician working in the NRCID serology laboratory stated that approximately 30% of its current assays on inpatients with acute or chronic hepatitis are positive for hepatitis C. However, most (80%) acute hepatitis cases in Mongolia appear to result from hepatitis A (L. Togooch, pers. commun.). A recent report by Japanese investigators gives a seroprevalence rate in 150 outpatients in Ulaanbaatar of 39% (hepatitis B) and 48% (hepatitis C). All participants in this study also had volunteered to participate in an epidemiologic study of hyperlipidemia at the Central Clinic Hospital in Ulaanbaatar (*10*). The incidence of hepatitis B is falling as a result of two major interventions begun in 1991. At that time, a national immunization program, including vaccination against hepatitis B, was started. In addition, a program to stop the reuse of phlebotomy needles and needles for injection was begun. Currently, all such needles are sterile, individually packaged, and disposable. Evidence attesting to the effectiveness of these interventions is emerging. Edstam et al. report a drop of hepatitis B carriage from a historical prevalence of 14% to 6.9% in a random cluster sampling of Mongolian 2-year old children. These encouraging results were found in both rural as well as urban settings with one dramatic exception. For unclear reasons, 40% of persons in the rural *aimag* Bayanhongor were positive when tested for hepatitis B surface antigen (*11*).

In spite of the overall encouraging decline in hepatitis B carriage, the impact of chronic hepatitis remains a major health problem for the country. Hepatocellular carcinoma is the most common malignancy in Mongolia (followed by gastroesophogeal, cervical, and lung cancers), and cirrhosis remains common enough to justify a 54-bed ward at NRCID dedicated to the care of patients with the disease. Although injection drug use appears to be rare, alcohol abuse is very common and almost certainly contributes to Mongolia’s problem with chronic liver disease (*12*).

Infectious disease physicians at the National Medical University and NRCID are well aware of the use of interferon for treatment of chronic hepatitis B and C but have been limited in utilizing it, primarily because of its expense. Only approximately 100 patients had been treated with interferon by 2001.

## Brucellosis

Approximately 23% of Mongolia’s population lives in rural areas and lead a nomadic or seminomadic way of life. Their diet is heavily dependent on meat and dairy products, reflecting the importance that large domesticated animals have played in the country’s history. In the past decade, the number of livestock has increased from 26 million to over 33 million, including 26 million sheep and goats, 3.8 million cattle, 3.1 million horses, and 350,000 camels (*13*). Not surprisingly, brucellosis remains one of the major veterinary and public health problems in Mongolia. The Brucella seroprevalence rate among cattle in 1987 ranged from 3.8% to 35% before a vaccination program (*14*) but now appears to be approximately 5%-10% with some focal areas close to 50% (Andrea Mikolon, pers. commun.). Seroprevalence in sheep and goats is less, approximately 2%. Nevertheless, *Brucella melitensis* appears to be the most common species of Brucella isolated from blood cultures taken from acutely ill patients (Andrea Mikolon, pers. commun.). In Mongolia, transmission to humans occurs primarily from direct contact with animals through injury while handling them or during slaughtering and to a lesser extent, from drinking contaminated milk (Andrea Mikolon, pers. commun.). As of 2001, approximately 8,000 human cases of chronic brucellosis were reported, and 1,000–1,500 new cases have been reported yearly since 1996 (*9*) (compared with approximately 100 cases annually in the United States [*15*]). Such a high caseload in this sparsely populated country is reflected in a 50-bed unit at NRCID dedicated to caring for people with this disease. Most patients on that unit appear to have chronic skeletal disease diagnosed by clinical features, x-ray findings, and positive serologic results. Cultures are rarely done because of lack of appropriate safeguards for this level III pathogen, but they may be performed occasionally at the University Central Laboratory of the National Medical University. Confirmation and speciation by using polymerase chain reaction (PCR) may also be obtained at the same facility. Treatment with doxycycline and gentamicin or rifampin is standard on the unit, but it is often administered for only 2 weeks rather than the minimal 6 weeks recommended in most recent reviews (*15*,*16*). Possibly, as a result, many of the inpatients have a history of relapsing disease and have been admitted for treatment on multiple previous occasions.

Attempts to control this enzootic infection have been unsuccessful because of an inconsistent strategy varying between vaccination of livestock and the destruction of infected animals. As of 2001, no uniform animal vaccination program existed in Mongolia. However, the World Health Organization has recently been conducting meetings with the Ministry of Health and the National Medical University to further assess the health impact of brucellosis in the country and make recommendations for its control.

## Sexually Transmitted Diseases

Sexually transmitted diseases (STDs) have become an increasing problem since Mongolia became fully independent of Soviet control in 1990. This increase may relate partly to temporary loss of central governmental control as well as a decreased economic base for the country. (The Soviet Union had provided 85% of developmental aid to Mongolia, amounting to 35% of the government’s annual budget [*2*]). Physicians at NRCID estimate that 600–1,100 female prostitutes lived in the country in 2001 and suggest, although data are incomplete, that this number represents an increase over the previous decade.

Reported cases of syphilis ranged from 705 to 810 annually during the years 1990 through 1996, but increased to 1,291 in 1997 and 1,329 in 1998. For most of the past decade, gonorrhea was reported in over 3,000 patients yearly except in 1990 (2,234 cases) and 1999 (2,207 cases) (9). These official statistics coincide with the perception of infectious diseases physicians of NRCID that the STD rate has increased since 1990. In addition, a 1997 report by Purevdawa et al. gives further support for this concern. The authors state that syphilis, gonorrhea, and trichomonas cases rate per 100,000 population increased from the mid-1980s (or 1993 in the case of syphilis) to 1995 as follows: syphilis, 18 to 32; gonorrhea, 51 to 142; and trichomonas, 47 to 155 (*17*). A recent study conducted in Ulaanbaatar among 260 patients attending a STD clinic found the prevalence of gonorrhea, chlamydia, and syphilis to be 31.1%, 8.1%, and 8.6%, respectively, for male patients and 10.3%, 9.9%, and 6.0% for female patients. Seventy-seven percent of female patients had trichomoniasis and nearly 20% of male patients had nongonococcal urethritis (*18*). Similar results were found in 110 women attending an STD clinic in Ulaanbaatar from whom genital samples obtained by insertion and immediate removal of tampons were tested by using PCR amplification. *Chlamydia trachomatis* (14%), *Neisseria gonorrhoeae* (11%), and human papillomavirus (HPV) (36%) were detected in the samples. Forty-four percent of the women with human papillomavirus had oncogenic genotypes detected (*19*).

In addition, antimicrobial drug resistance has become common in *N. gonorrhea* isolates. Forty-eight percent to >50% have been found to be resistant to penicillin; close to 15% are resistant to tetracycline, and nearly 25% are resistant to ciprofloxacin (*20*,*21*).

Approximately 20,000 patients have been screened for HIV infection at NRCID every year since 1987. The laboratory uses a microtiter agglutination system obtained from Japan as a rapid screen and enzyme-linked immunosorbent assay (ELISA) from either Japan or Italy as second-level tests on samples found positive by using the screen. As of 2001, two patients were positive: one acquired HIV in another country, and the second is thought to have acquired HIV through contact with an African person visiting Mongolia. Nevertheless, the increased rates of STDs since the mid-1980s, in addition to increased tourism in the country, raise concern that HIV will become a problem in Mongolia and justify the public health education initiatives already taking place.

## Respiratory Tract Infections, Infectious Causes of Diarrhea, and Parasitic Infections

Respiratory tract infections and diarrhea are very common in Mongolia, especially among children. Generally, respiratory illnesses including pneumonia are most common during the winter and, at least in 1991, accounted for most the country’s infant mortality rate (*2*). Officially, the infant mortality rate in 1990 was 64.4 per 1,000 live births and decreased to 37.3 per 1,000 live births in 1999 (*22*).

Infectious diarrhea, on the other hand, is more problematic during the short summer season. Laboratories in the major hospitals of Ulaanbaatar including NRCID identify enteric bacterial pathogens by using manual methods. The most commonly identified pathogens have been *Shigella flexneri* and *Salmonella* species such as *S. enteritidis*Typhimurium (D. Regzedmaa, pers. commun.). However, supplies and equipment necessary to isolate and identify *Campylobacter* species*,* enterotoxigenic and enterohemorrhagic *Escherichia coli,* and rotavirus are not available; consequently, information regarding the prevalence of these organisms is not available.

An outbreak of cholera involving approximately 100 persons occurred in 1996 but was rapidly brought under control. No cases have been identified since that time, and the source of the outbreak remains uncertain. Typhoid fever appears to be uncommon. At NRCID, one case has been recognized since 1999. In that instance, a 42-year-old man with fever, diarrhea, and a perforated small bowel required resection after he had treated himself before hospitalization with ampicillin and gentamicin.

Official statistics show a gradual decline in reported cases of salmonellosis and dysentery from 866 and 1,930 cases, respectively, in 1990, to 243, and 1,383 cases in 1999 (*9*). Nevertheless, infectious diarrhea remains a major health problem during the summer months in Ulaanbaatar and a 34-bed unit at NRCID is dedicated to the care of patients with this disease.

The director of the enteric parasite laboratory at NRCID reported that *Enterobius* *vermicularis* constituted approximately 90% of detected intestinal parasites. The remainder consisted of *Ascaris lumbricoides* and *Taenia* species. This finding corresponds with a recent survey of rural residents near Ulaanbaatar which found that enterobiasis and hydatidosis were the two major helminthic infections in Mongolia. Lee et al. offered the speculation that soil-transmitted parasites such as *A. lumbricoides* are less common because soil-derived vegetables are uncommonly included in the typical Mongolian diet (*23*).

Most households in rural Mongolia own livestock (sheep, goats, horses, cattle) and over 50% own dogs. Not surprisingly, human infection with *Echinococcus granulosis* is seen by physicians in Ulaanbaatar, although less frequently than in the neighboring northern provinces of China. A recent survey conducted in northwest Mongolia detected a seroprevalence of 5% by using an ELISA on 334 persons (*24*). An earlier report by Davaatseren, et al. claimed echinococcus was the cause for 18% of the surgical cases in the First Clinical Hospital of Ulaanbaatar in 1993 (*25*).

## Other Infectious Diseases

Cases of plague, due to infection with *Yersinia pestis,* have been seen in approximately 40 patients each year, especially in rural Mongolia. Transmission appears usually to occur as a result of hunting marmots (*Marmota sibirica)*, large rodents especially plentiful throughout the vast steppes of Central Asia. A recent case of pneumonia occurred in Mongolia’s western-most province (*aimag*), Bayan Olgii; the 93 persons who had contact with the patient received prophylactic tetracycline. Enzootic plague also may be maintained in the Mongolian gerbil (*Meriones unguiculatus)* and its flea (*Nosopsyllus laeviceps)* (*26*).

Occasional cases of tularemia have been reported in Mongolia. Animal cases of anthrax involving cattle, sheep, and goats are reported sporadically in the country. Occasional human cases of cutaneous or intestinal anthrax also have been reported. An animal vaccine is available but has not been administered as part of a nationwide program.

Bovine spongioform encephalopathy is unknown in Mongolia and is not anticipated because all cattle are strictly pasture-fed. On the other hand, foot-and-mouth disease caused by apthovirus of the family *Picornaviridae,* although thought to be eradicated in 1973, reappeared along the Chinese border in the year 2000. More recently, it spread from the eastern Mongolian provinces and by March 2001 appeared among the livestock on the outskirts of Ulaanbaatar. Prompt intervention including livestock vaccination brought the outbreak under control, and most districts around the capital city had their quarantine lifted by May 2001.

Rabies remains an endemic problem especially among dogs and wolves, with occasional human cases reported. Although rabies immunoglobulin and human diploid cell vaccine are not available, a locally produced, goat brain–derived vaccine is available for use in Mongolia.

Lyme disease is not reported in the country, but a recent study in northeastern China (Inner Mongolia) documented *Borrelia garinii* and *B. afzelii* in *Ixodes persulcatus* ticks (*27*). Therefore, the possibility exists of human disease occurring in Mongolia. Tick-borne encephalitis due to a flavivirus infection and hemorrhagic fever with renal syndrome, both present in Russia, are not recognized in Mongolia (*28*).

## Antimicrobial Drug Resistance

Antimicrobial drugs generally available at NRCID include penicillin G, ampicillin, oxacillin, cephalosporins, including ceftriaxone and cefotaxime, gentamicin, chloramphenicol, ciprofloxacin, tetracycline, erythromycin, trimethoprim-sulfamethoxazole, rifampin, isoniazid, ethambutol, and pyrazinamide. Aminoglycoside blood levels were not routinely available, as of 2001, although they could be obtained through private specialty laboratories in Ulaanbaatar.

Oral antimicrobial drugs have been preferred over those administered parentally, whenever possible. Sterile intravenous infusion solutions are available and prepared locally at each major hospital in Ulaanbaatar. However, intravenous catheters are not readily available, which necessitates that intravenous antimicrobial drugs be given through sterile needles. These needles are promptly removed after each infusion. As of 2001, antimicrobial drugs are commonly prescribed for at least 3 postoperative days to patients undergoing even minor surgery (such as simple lipoma removal) to reduce the incidence of postoperative wound infections.

Each major hospital in Ulaanbaatar has its own bacteriology laboratory, where organisms are identified by using manual methods and susceptibilities are determined by using antimicrobial drug disks. Nevertheless, antimicrobial drug susceptibility profiles appear to be unavailable. Several hospital laboratories could not, for example, provide information regarding their prevalence of methicillin-resistant *Staphylococcus aureus* (MRSA). The director of the The World Health Organization (WHO)-sponsored PCR section of NRCID central laboratory reported a rate of 20% to 30% MRSA. However, no details or written reports were available for review.

## Hospital Infection Control

Sinks and bar soap are available throughout all hospitals. Individual paper towels were not present, however, and reusable cloth towels were used in their place. Healthcare providers, as a rule, did not wash their hands between examining patients during rounds; rather, hand washing generally was done only at the end of rounds after all patients had been seen.

## Immunization

Mongolia’s current national immunization program began in 1991 and includes vaccines against TB, polio, hepatitis B, diphtheria, pertussis, tetanus, and measles. As of 2001, the vaccination rate for all children in the country was 98%. Immunization against rubella was to be added to the official program during 2002. Mumps vaccine is not on the official vaccination schedule, however. Of interest, an outbreak of mumps occurred in Ulaanbaatar during the spring of 2001. Mongolia did not experience the outbreak of diphtheria in the 1990s as Russia did (*28*). Four cases were reported for the country in 2000. WHO certified that Mongolia was free of polio in 2000. Immunization against influenza is sporatic and dependent on outside funding. (L. Togooch, pers. commun.).

## Summary

Since 1990, Mongolia’s health system has been in transition. Impressive gains have been accomplished through their national immunization program, started in 1991. Of particular note is the decreased incidence of acute hepatitis B. Nevertheless, the country continues to struggle with chronic hepatitis B- and C-related liver failure and hepatocellular carcinoma (exacerbated by alcoholism, an additional common social health problem in Mongolia). Three other major infectious disease problems include brucellosis, TB with significant single drug resistance being identified, and rising rates of STDs. Respiratory illnesses including pneumonia constitute a major cause of infant deaths, especially during the prolonged winters, and infectious diarrhea remains a common public health problem during the summers.

As of 2001, only two HIV infections have been detected in Mongolia. Nevertheless, public health leaders remain concerned that its incidence may soon increase. Other infectious diseases of importance include echinococcus, plague, tularemia, anthrax, foot-and-mouth, and rabies.

Antimicrobial drugs are available to the public without prescription and also may be given excessively to postoperative patients. Antimicrobial drug–susceptibility trends generally are not available at the major teaching hospitals in Ulaanbaatar. Infection control policies, especially handwashing, appear to need more attention.

## Challenges

We conclude with our recommendations emphasizing areas for focus and further development, which would seem most applicable and potentially beneficial over the next 10 years. Further progress in transitioning from the clinical diagnosis of TB to diagnosis confirmed by widespread availability of culture for *Mycobacterium tuberculosis* (with accompanying antimicrobial drug–susceptibility results) will be a great help in directing limited resources and optimizing individual treatment programs by using DOT. Given the high prevalence of chronic viral hepatitis (and for other reasons related to social and economic development issues), a national program that reduces individual excessive alcohol consumption through education and treatment centers is appropriate. A national, consistently applied program to reduce the prevalence of Brucella infection of animal livestock is needed. Continued emphasis on public health measures to reduce the spread of sexually transmitted diseases and prevent the incursion of HIV is of great importance. A national program for control of antimicrobial drug use based upon physician prescription rather than general public access is necessary. A system of consistently performing susceptibility studies on common bacterial isolates with subsequent publication of antimicrobial drug–susceptibility trends, at least in the referral hospitals in Ulaanbaatar, would be an important advance. Hospital infection control, including handwashing between individual patient examinations and antimicrobial drug use policies, needs to be further developed in Mongolia. Expansion of the excellent national immunization program to include rubella, mumps, influenza, and possibly hemophilus vaccines would provide additional benefit to the people of Mongolia.
